# Neuron-targeted Knockout of APE1 Forces Premature Cognitive Impairment and Synaptic Dysfunction in Adult Mice

**DOI:** 10.14336/AD.2022.0331

**Published:** 2022-12-01

**Authors:** Ling Zhu, Sulaiman H. Hassan, Xuguang Gao, Joycelyn Q. Johnson, Yangfan Wang, M. Victoria Bregy, Zhishuo Wei, Jun Chen, Peiying Li, R. Anne Stetler

**Affiliations:** ^1^Pittsburgh Institute of Brain Disorder & Recovery and Department of Neurology University of Pittsburgh School of Medicine, Pittsburgh, PA 15213, USA.; ^2^Geriatric Research, Education and Clinical Center, Veterans Affairs Pittsburgh Health Care System, Pittsburgh, PA 15261, USA.

**Keywords:** DNA repair, DNA damage, apurinic/apyrimidinic endonuclease-1, aging

## Abstract

Adaptable and consistent neural function relies at least in part on the ongoing repair of oxidative damage that can accumulate in the brain over a lifespan. To determine whether forebrain neuron-targeted knockout of AP endonuclease 1 (APE1), a critical enzyme in the base excision DNA repair pathway, contributes to neuronal impairments, we generated APE1 conditional knockout mice under the control of the CamKIIα promotor (APE1 cKO). Spatial learning and memory were tested using the Morris water maze. Synaptic markers, including synapsin, vGLUT, GABA1, and GAD were immunostained and quantified. Dendritic morphology and number were characterized using Golgi staining. Long-term potentiation (LTP) was measured in slices from the 6-month-old brain. APE1 cKO mice did not significantly differ from WT mice in the learning phase of the Morris water maze, but performed significantly worse during the memory phase of the Morris water maze. vGLUT, GABA1, and GAD immunostaining was significantly decreased in APE1 cKO mice without concomitant changes in the number of synapsin-positive structures, suggesting that neural networks may be impaired but not at the level of total presynaptic structures. Dendrites were reduced both in number and length of spines in APE1 cKO mice. APE1 cKO brain slices exhibited decreased LTP induction compared to WT brain slices. Together, these data indicate that the conditional loss of APE1 in forebrain neurons leads to a phenotype consistent with expedited brain aging

Aging-related cognitive impairments are emerging as a global health concern with the increase in human lifespan [1, 2], emphasizing the need to understand both normal and pathological effects of age on cognitive tasks. Cognitive function declines steadily with aging, with or without amyloid deposition [3, 4], and recent evidence suggests that aspects of age-related cognitive decline may begin in healthy adults as early as the third decade of human life [5]. Impaired cognition decreases the quality of life for both the patient and their family [6, 7]. Therefore, combating age-related cognitive impairment is an important and timely research topic.

During normal aging, pathological changes appear gradually and are often associated with prolonged events such as sustained inflammation and oxidative stress [8-11]. The failure of neurons to adapt to age-related stress results in neuronal dysfunction, including decreased synaptic transmission [12, 13], dendritic spine loss [14] and suppressed neuronal network activity [15]. These and other factors are thought to underlie aging-related cognitive impairment. As an example, DNA damage is a significant consequence of oxidative stress and is closely associated with aging-related cognitive decline [10, 16]. Although endogenous mechanisms exist to repair DNA damage, these repair mechanisms become less effective with aging, leading to an accumulation of DNA lesions, loss of genome stability, and altered gene expression [17]. Such alterations in DNA structure may contribute to age-related neurodegenerative diseases [18] and cognitive dysfunction [19-21].

The enzyme apurinic/apyrimidinic endonuclease-1 (APE1) participates in both the base-excision repair (BER) pathway, one of the main DNA repair pathways responsive to oxidative DNA damage, as well as in transcription and cellular functions through its redox activity [22]. Overexpression of APE1 protein in cultured cells or animals is neuroprotective against oxidative damage and ischemic brain injuries [23-25], whereas conditional knockout of APE1 exacerbates ischemic brain injury [26]. Polymorphisms in DNA BER enzymes, including APE1, are associated with age-related cognitive impairments in healthy elderly individuals and reduced performance on mini-mental state examinations in AD patients [27, 28].

In the current study, we generated calcium/calmodulin-dependent protein kinase II alpha (CaMKIIα) promotor-driven APE1 knockout mice, in which Cre recombinase promoter activity deletes APE1 in neurons of the forebrain, including in the selectively vulnerable CA1 pyramidal cell layer of the hippocampus [29, 30]. Although many regions of the brain may be susceptible to aging-related changes in either structure or function, the somatosensory cortex and hippocampus have been well studied in terms of both cellular and associated functional changes related to aging, including loss of dendritic spines [31-33], as well as high CaMKIIα promotor activity [30]. Targeted knockout of APE1 allowed us to attribute aging-related changes in neuronal function to the absence of APE1 in CaMKIIα-expressing neurons, which are enriched in the cortex and hippocampus, and their associated behavioral and functional outcomes.

## MATERIALS AND METHODS

### Generation of and breeding of mutant mice

APE^fl/fl^ mice were generated and characterized as previously described [26]. To achieve APE1 gene knockout predominantly in forebrain neurons, we crossed APE^fl/fl^ mice with a Camk2a-cre transgenic line expressing Cre recombinase under the control of the calcium/calmodulin-dependent protein kinase II alpha (CaMKIIα) promotor (Stock No. 027310, The Jackson Laboratory). Expression controlled by this promotor is enriched in neurons in the CA1 pyramidal cell layer in the hippocampus as well as the dentate gyrus and approximately one-third of the neurons in layers II/III and VI of the neocortex, and to a lesser extent in subcortical regions [30] [34]. These mice will be referred to throughout the paper as APE cKO mice. As genotype controls, we used littermate mice lacking Cre recombinase expression (referred to as WT controls). All mice were on a C57BL/6J genetic background. Only male mice (n=50) were used for this study in order to properly power the statistical analyses, draw on the extensive literature in aging males, and, for this initial study, avoid confounds of hormone fluctuations in aging female mice.

All animal procedures were approved by the Institutional Animal Care and Use Committee at the University of Pittsburgh, and in compliance with the National Institute of Health guidelines. The animal data reporting of the current study follows the ARRIVE 2.0 guidelines [35].

### Golgi staining

Golgi staining was performed using the FD Rapid GolgiStain™ kit (FD NeuroTechnologies, Columbia, MD), according to the manufacturer’s instructions. Briefly, fresh brain samples were immediately prepared in the impregnation solution for 2 weeks followed by immersion in solution C for 1 week at room temperature. Brain tissues were sliced into 100 µm-thick coronal sections and mounted on gelatin-coated slides. The approximate stereotactic coordinates were as follows: layers II/III of the somatosensory cortex (ML 2.20, AP -1.5, DV 0.6), hippocampal CA1 (ML 1.7, AP -2.0, DV 1.4), and hippocampal CA3 (ML 2.0, AP -2.0, DV 2.2). After rinsing in distilled water, slides were immersed in freshly prepared staining solution for 10 minutes followed by dehydration in ethanol and clearing in xylene. Dendritic spines were quantified as spine length and number of spines per 10 µm length of spine. From each area, 4-5 neurons were counted per animal, and 4-5 animals were used per genotype. Images were captured using an Olympus BX51 microscope at 4X, 10X, and 60X magnification. Average spine densities were defined as the number of protrusions divided by the length of dendritic shaft and were measured using the ImageJ software.

### Immunofluorescence staining

Immunofluorescence staining was performed on 25-μm free-floating coronal brain sections as previously described [26, 36]. For reference, approximate stereotactic coordinates were as follows: layers II/III of the somatosensory cortex (ML 2.2, AP -1.5, DV 0.6), hippocampal CA1 (ML 1.7, AP -2.0, DV 1.4), and hippocampal CA3 (ML 2.3, AP -2.0, DV 2.3). Primary antibodies included the following: anti-APE1 (ab268072, abcam), anti-vGlut-1 (75-066, NeuroMab), anti-GABA-A-R-Alpha1 (GABRA1) (75-136, NeuroMab), anti-synapsin (ab1543p, Millipore), anti-GAD (ab1511, Millipore), anti-NeuN (ABN78, Sigma Aldrich).

### Morris water maze

The Morris water maze test was performed at 4, 5, and 6 months of age, as previously described [23, 37]. Briefly, a circular platform (11 cm in diameter) was submerged in one quadrant of a circular pool (109 cm in diameter) of opaque water. Each mouse was put in the water in one of four quadrants and was allowed up to 60 seconds to find the platform. When each trial ended, the mouse was placed on the platform for another 30 seconds to help it remember the external spatial cues displayed around the room. During the learning phase, the time needed to find the submerged platform (latency) was recorded for every trial. Four trials per day were performed for four consecutive days. A single 60 second probe test was performed on the final day of testing, wherein the platform was removed (memory) and the time spent in the goal quadrant was recorded. Data were recorded and analyzed using AnyMaze Software (Stoelting Co., Wood Dale, IL). All behavioral tests were carried out by researchers who were blinded to group allocation.

### Two-object novel object recognition

The novel object recognition (NOR) task was performed to evaluate recognition memory [38, 39]. On days that the NOR and MWM tasks overlapped, the NOR was executed first. Briefly, the task was conducted in an open field arena with two objects placed in the center area of the arena. The objects were of different shapes and appearance, but similar height and volume. On the day before testing, mice were habituated once in the empty open field by being initially placed facing the wall nearest to the experimenter and allowed to explore the open field for 5 minutes. On the testing day, two identical objects were placed in the open field, 5 cm away from the wall, and the mouse was allowed to move freely for familiarization with 20 seconds of exploration of both objects, then recorded for a 10-minute observation period. Object exploration was scored using videography when the mouse contacted or sniffed the object or approached the object (i.e., when the distance between the snout and the object was less than 2 cm). The objects and the open field were cleaned with 70% (vol/vol) ethanol to minimize olfactory cues. During the novel cue testing period (occurring 1 hour following the observation period), one object was replaced with a novel object (differing in shape and contrast color but placed in the same location) and the mouse was recorded for a 10-minute testing period. The discrimination index was defined as the exploration time of the novel object (as assessed by the AnyMaze automated video-tracking system) divided by the total exploration time.

### Electrophysiology (Long term potentiation)

Mice were sacrificed with carbon dioxide inhalation and decapitated. Brains were quickly removed and transferred to oxygenated, ice-cold artificial cerebrospinal fluid (aCSF) with high magnesium (124 mM NaCl, 26 mM NaHCO_3_, 10 mM glucose, 3 mM KCl, 1.25 mM KH_2_PO_4_, 5 mM MgSO_4_, and 3.4 mM CaCl_2_). The brains were cut into 400 μm coronal hippocampal slices using a tissue chopper (Thermo Scientific Microm HM450, Waltham, Massachusetts, U.S) and maintained for 30 min in a recovery chamber with oxygenated aCSF at RT. The hippocampal slices were then transferred to an interface recording chamber and exposed to a warm, humidified atmosphere of 95% O_2_/5% CO_2_ with preheated (33 ± 0.5 °C) oxygenated aCSF perfused at 1 mL/min. After incubation in the recording chamber for 1 hour, a single glass pipette filled with normal aCSF was used to record field excitatory postsynaptic potentials (fEPSPs). Before each experiment, the stimulation intensity was adjusted to 40-50% of the maximum of fEPSP without spike component, which was considered the test pulse. Next, the test pulse was delivered at a 0.05Hz and the responses were recorded. After 10 mins of baseline stimulating, LTP was induced and the fEPSP was analyzed for its initial falling slope [40].

### Image Processing

Images were acquired using confocal microscopy (60x magnification with z-stacks), acquiring one image in each matched anatomical region (CA1, CA3, DG, and CTX) per animal, and processed with Image J or IMARIS for unbiased counting of automatically recognized cells or synaptic spots by a blinded observer. Excitatory and inhibitory synapses were imaged and processed with Image J and three-dimensional reconstruction using IMARIS software (Bitplane). Using IMARIS tools such as surface rendering, fluorescence thresholding, and masking of unwanted immuno-labeling, we were able to obtain a representation 3D structure of an individual neuron including the number and location of its glutamatergic and GABAergic synaptic inputs [41]. Finally, the spots were quantified by imaging software as the number of positive spots per mm^2^.

### Statistical Analysis

Data were expressed as mean±SEM and analyzed for normality using the Shapiro-Wilk test. Students’ t-test was used to measure between-group differences and non-parametric tests were used for non-normally distributed data. Behavioral data were analyzed using two-way ANOVA for multiple comparisons followed by the Bonferroni post hoc. LTP timecourse was analyzed using two-way ANOVA followed by the Tukey post hoc. Statistical analysis and image plotting was conducted by using GraphPad Prism 8.0. A *p* value less than 0.05 was defined as statistically significant.

**Figure 1.**
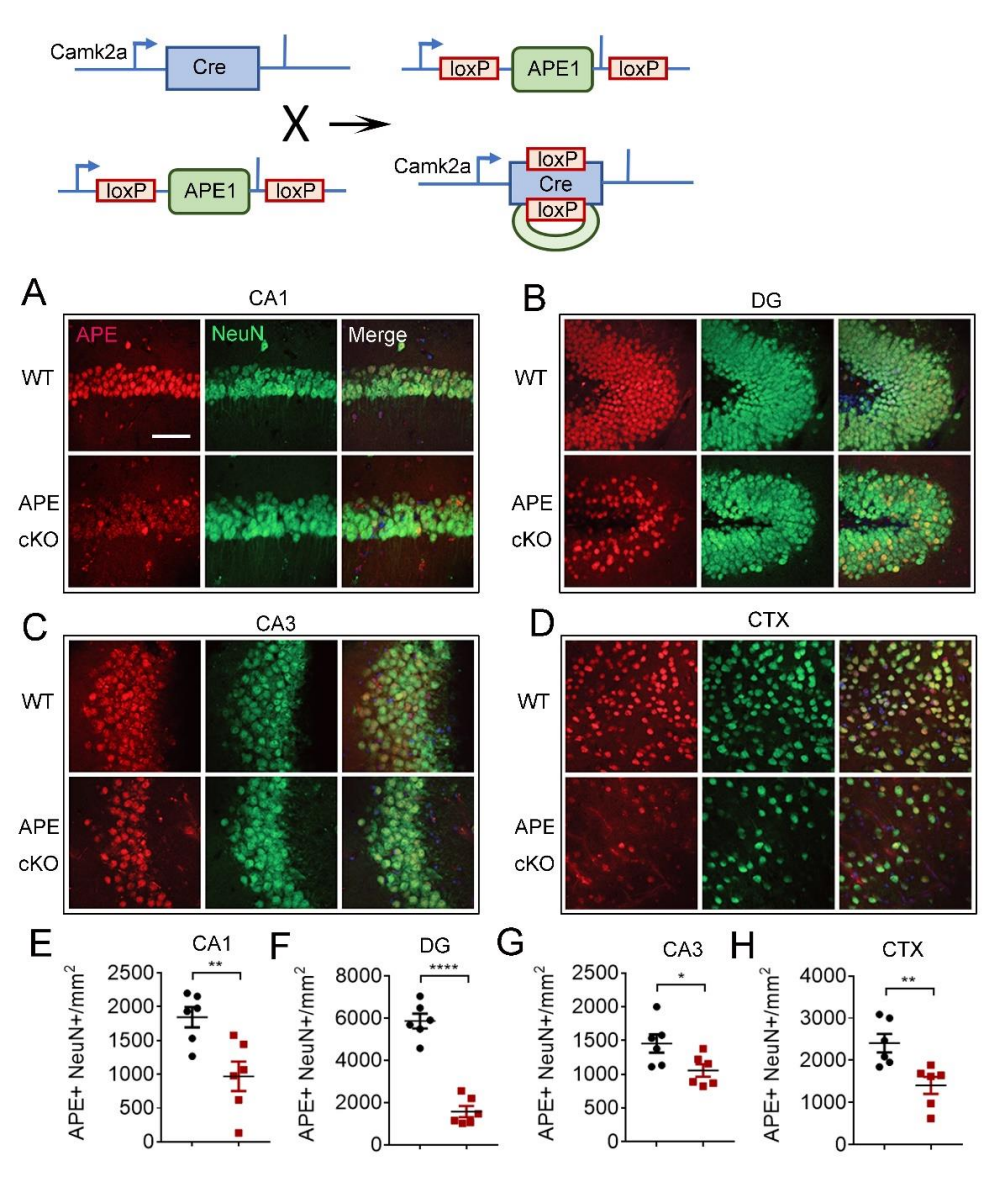
CaMKIIα-driven conditional knockout of APE1 (APE1 cKO) leads to significantly reduced APE expression in mature neurons of cortex and hippocampus. Mice with CaMKIIα promoter-driven conditional knockout of APE1 were generated by breeding APE1^flox/flox^ mice with hemizygous CaMKIIα-cre mice, yielding CaMKIIα-Cre:APE1flox/flox mice. Immunofluorescence staining showed lack of localization of APE expression with NeuN+ cells (mature neurons) in cKO brain. Representative images of APE (red) and NeuN (green) in CA1 (A), DG (B), CA3 (C) and CTX (D). Quantification of double staining of APE1 and NeuN in CA1 (E), DG (F), CA3 (G) and CTX (H). Bar=50 µm. All data are presented as scatterplots with mean±SEM, n=6 animals/group. *P<0.05, **P<0.01 analyzed by Student’s t-tests.

**Figure 2.**
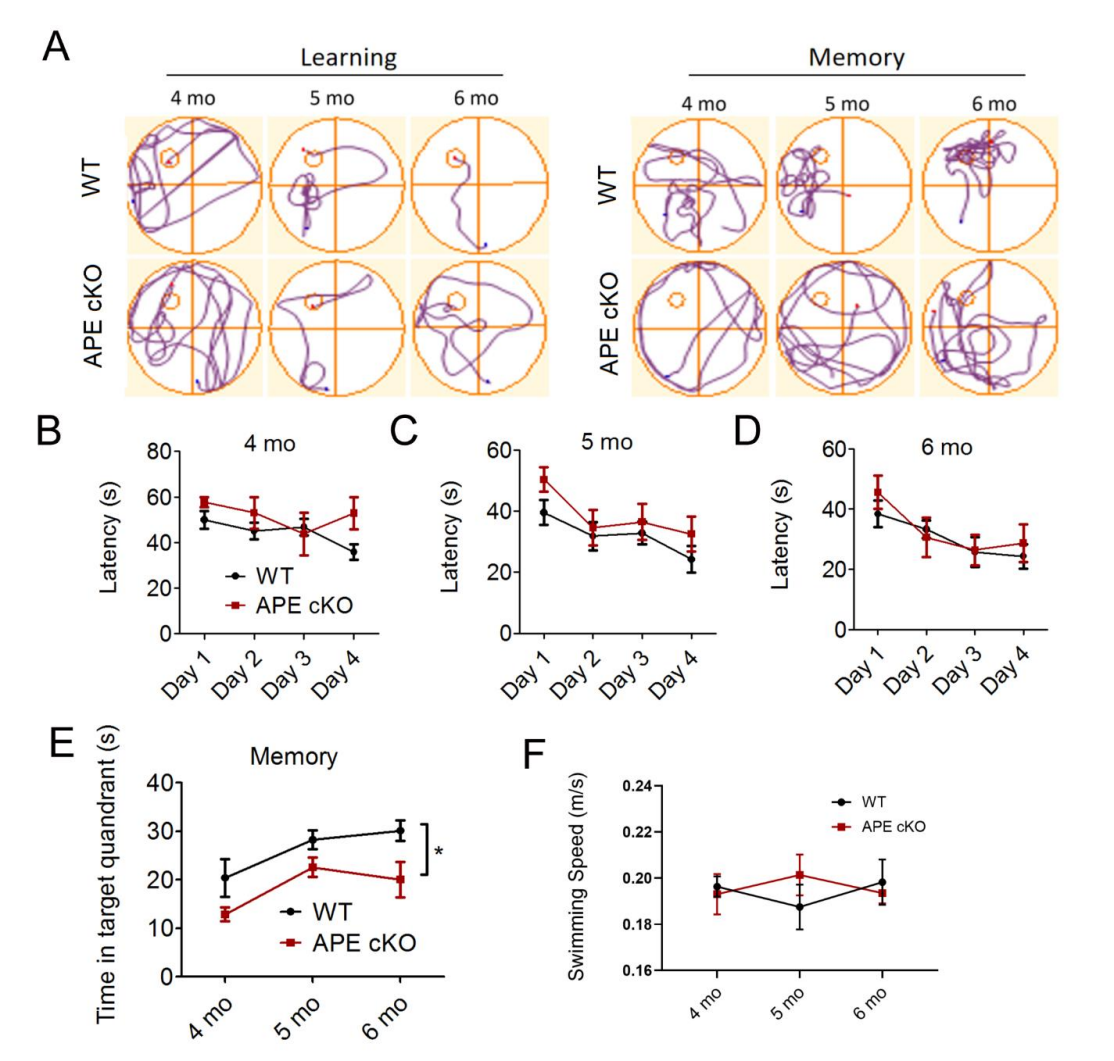
Deficiency of APE1 results in impaired spatial memory function at 4-6 months of age. (A-F) The Morris water maze was conducted at 4, 5 and 6 months of age in WT or APE cKO mice. (A) Representative swim path traces of mice attempting to find an underwater platform (learning) or searching for the platform after its removal (memory). Latency (time needed to find the platform) was recorded from day 1 to day 4. Time in target quadrant reflects memory and was recorded on day 5. APE cKO mice did not differ in latency to find the platform at 4 months (n=13 WT, 5 APE cKO) (B), 5 months (n=14 WT, 12 APE cKO) (C), and 6 months (n=10 WT, 9 APE cKO) (D) of age, but spent less time in the target quadrant when the platform was removed (n=5 WT, 6 APE cKO) (E). (F) Swim speeds were measured on day 5 to ensure swimming abilities were comparable between the two groups. *P<0.05; **P<0.01 vs matched wild type mice, analyzed by two-way ANOVA followed by Bonferroni post-hoc. All data shown as scatterplots with the mean±SEM.

## RESULTS

### CaMKIIα-APE1 cKO leads to reduced expression of APE1 in forebrain neurons

The CamKIIα promotor can drive GFP expression in about 70% of granule and pyramidal neurons in the hippocampus and about 30% of neurons in the neocortex [29]. We first examined whether conditional knockout of APE1 by CaMKIIα-Cre reduced the expression of the DNA repair enzyme APE1 in the hippocampus and cortex by co-immunostaining APE1 and the mature neuron marker, NeuN. As NeuN is not exclusively localized to CaMKIIα-expressing cells and a heterogenous population of mature neurons exist in different brain structures, one would not expect complete knockout in all NeuN^+^ cells. Accordingly, we found that the number of APE1^+^NeuN^+^ cells per mm^2^ in the hippocampus and cortex was significantly reduced in CA1, CA3 and DG subregions of the hippocampus (Fig. 1A-C, E-G). The most dramatic reduction of APE1+/NeuN+ cells was observed in the DG, consistent with a wide range of prior studies that indicate the DG has a high number of CaMKIIα-expressing neurons [30]. Similarly, APE1 expression in NeuN^+^ cells was also reduced in layers II/III of the somatosensory cortex (Fig. 1D, H) of APE cKO mice compared to WT mice.

**Figure 3.**
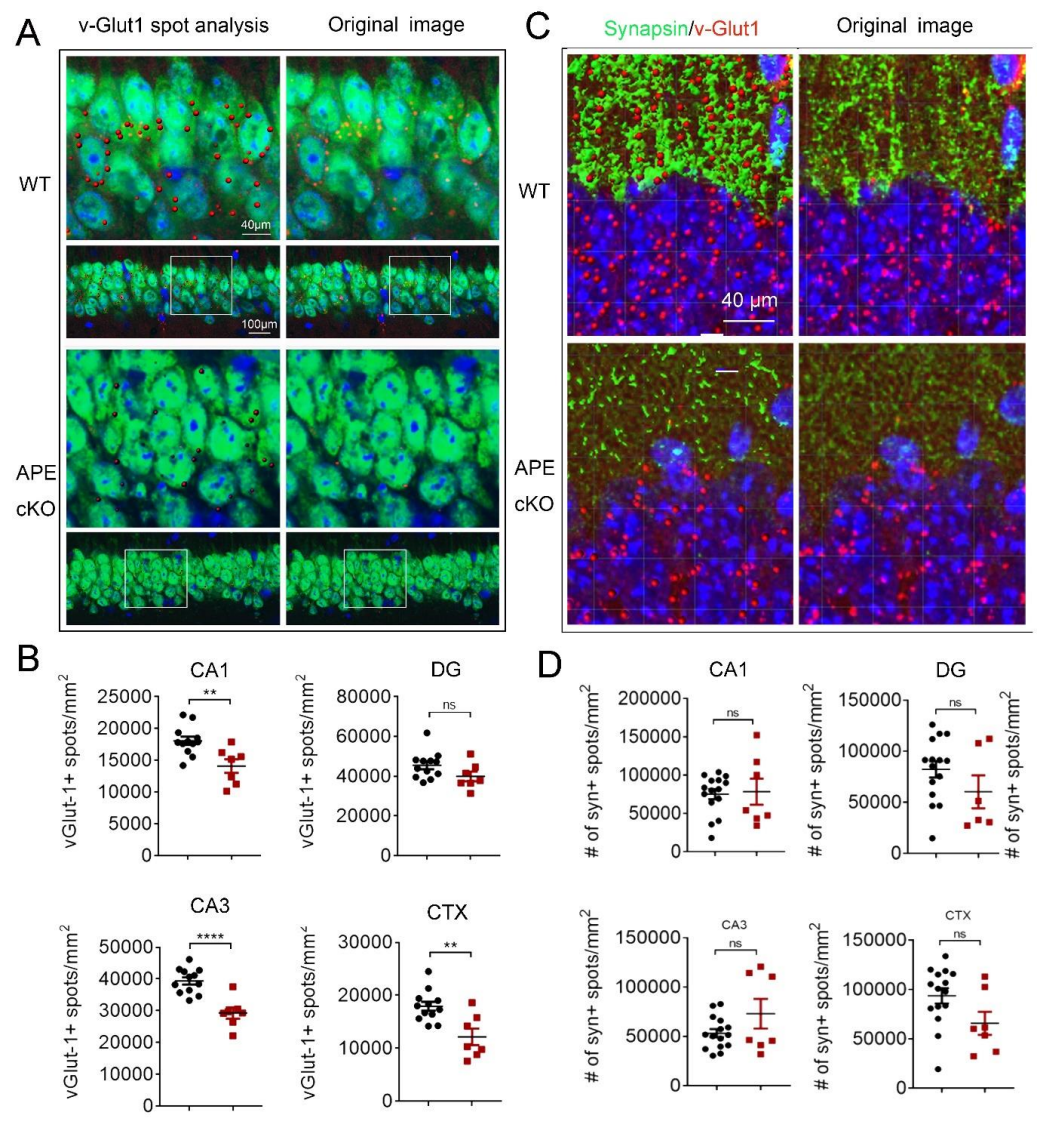
Double immunofluorescence for vGlut1-NeuN and vGlut1-Synapsin in the hippocampus and cortex. (A) Representative images demonstrating vGlut1 (red) and NeuN (green) immunofluorescence staining in CA1 and cortex (CTX). Scale bar: 50 µm. (B) Quantification of vGlut1-positive spots in CA1, dentate gyrus (DG), CA3, and cortex (CTX). (C) Representative images demonstrating vGlut1 (red) and Synapsin (green) immunofluorescence staining in CA1, DG, CA3, and CTX of the mice brain. (D) Quantification of synapsin-positive spots in CA1, DG, CA3. and CTX. All data shown as scatterplots with the mean±SEM, n=6 animals/group. *P<0.05, **P<0.01, ***P<0.001 analyzed by Student’s t-tests.

### CaMKIIα-driven conditional APE1 knockout promotes neurological dysfunction

In order to examine whether a deficiency in APE1 impairs cognitive function at an earlier stage of life compared to WT littermates, we subjected WT and APE1 cKO mice to the Morris water maze, a spatial learning and memory task, at 4-6 months of age [37]. We found no difference between WT and APE cKO mice in the latency to locate the submerged platform at 4, 5 or 6 months of age (Fig. 2A-D). However, the ability to remember the location once the platform was removed (memory phase) was significantly impaired in APE1 cKO mice compared to WT mice (Fig. 2A, E). To ensure the results were not confounded by impairments in swimming, we examined the swim speed of both groups and found no differences between genotypes in any of the four quadrants of the pool, during either the learning or memory phase of the test (Fig. 2F). The novel object recognition test also helps to measure recognition memory in rodents. We found that the discrimination index (i.e, the time spent exploring the novel object divided by the total time exploring both familiar and novel objects) displayed a statistical trend (p=0.35, Mann-Whitney U test, n=7/group) toward a decrease in the APE1 cKO mice compared to WT mice (data not shown). As these two memory tasks are distinct, the novel object recognition task may require longer aging periods to reveal statistically significant differences between groups or may have been underpowered.

**Figure 4.**
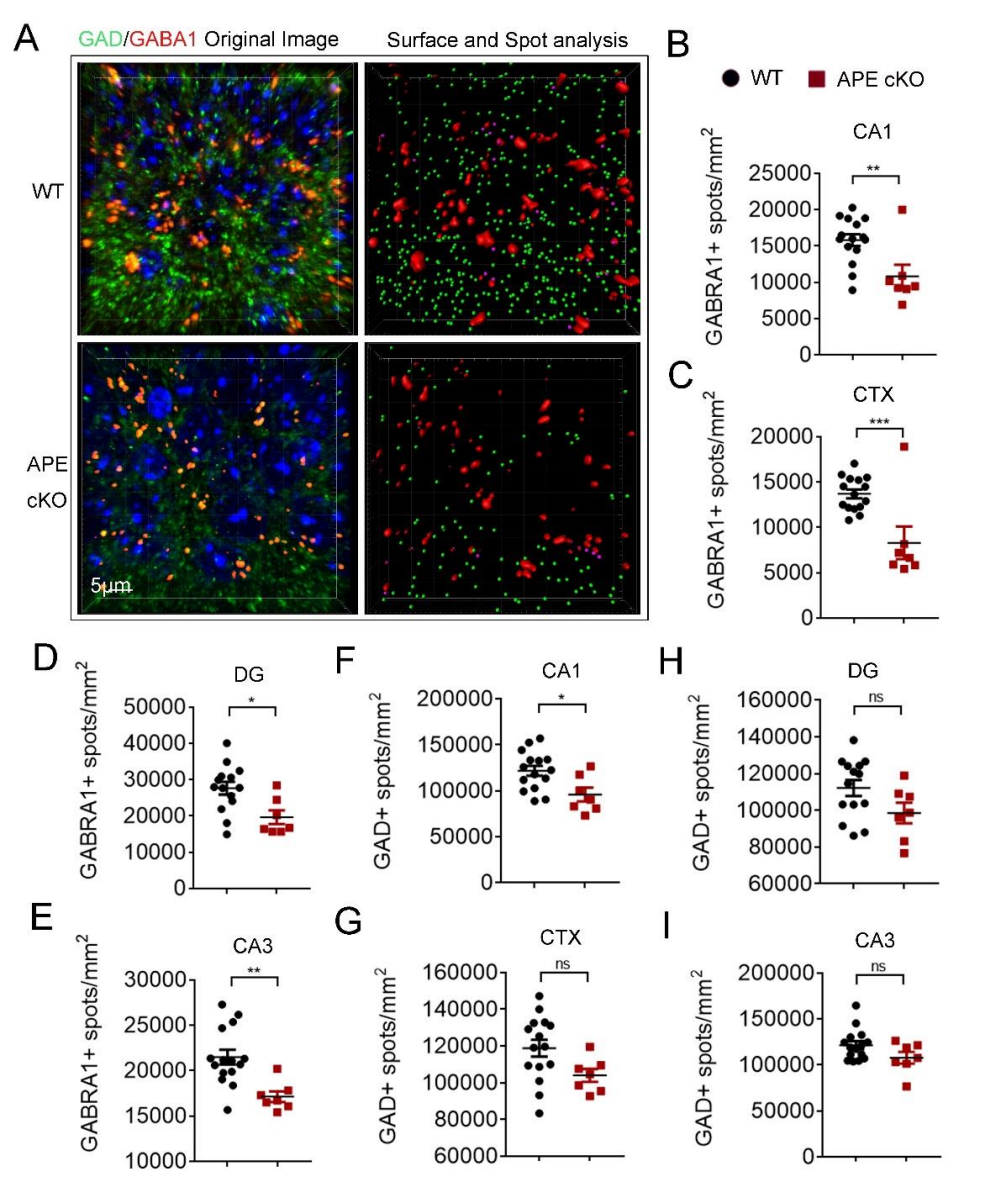
Reduced expression of GABRA1 and GAD in APE1 cKO brain. (A) Representative images demonstrating GABRA1 (red) and GAD (green) immunofluorescence staining in CA1, dentate gyrus (DG), CA3, and cortex (CTX). Scale bar: 50 µm. (B-E) Quantification of GABRA1-positive spots in CA1 (B), CTX (C), DG (D) and CA3 (E). Quantification of GAD-positive spots in CA1 (F), CTX (G), DG (H), and CA3 (I). All data shown as as scatterplots with the mean±SEM, n=6 animals/group. *P<0.05, **P<0.01,***P<0.001 analyzed by Student’s t-tests.

### CaMKIIα-driven conditional APE1 knockout potentiates synaptic network loss in brain

To explore cellular changes associated with the early onset of cognitive decline in APE1 cKO mice compared to WT mice, we examined synapses of neuronal networks in the hippocampus and layers II/III of the somatosensory cortex at 6 months of age. We first examined the expression of synapsin, a panneuronal marker of synapses, to determine if APE1 cKO led to gross changes in the number of synapses. We found that the number of synapsin immunostained spots did not change significantly between the APE1 cKO and WT mice in any of the observed brain regions (Fig. 3C, D), indicating that the density of synpasin-positive synapses does not appear to be altered at this early timepoint. To examine an excitatory synaptic marker relevant to learning and memory, we found that the expression of vGLUT1, an excitatory glutamatergic pre-synaptic protein necessary for the packaging of glutamate into presynaptic vesicles, was significantly reduced in CA1 and CA3 of the hippocampus and the cortex of the APE1cKO mice compared to WT mice, based on IMARIS quantification of the positive spots of vGLUT1 (Fig. 3A, B). However, no significant changes between genotypes were observed in the quantification of vGLUT1 positive spots in the DG of the hippocampus (Fig. 3A, B), possibly reflective of the glutamatergic inputs from the entorhinal cortex that may not express CamKII, and thus still maintain endogenous APE1 expression (as searched on the MGI recombinase activity database).

As GABAergic synaptic transmission is an important inhibitory neuronal activity, we also examined the impact of APE1 cKO on the expression of GABAergic synaptic markers, GABRA1, a postsynaptic protein, and glutamic acid decarboxylase (GAD), a pre-synaptic protein [42]. We found that the number of GABRA1 and GAD positive spots in the brain of APE1 cKO mice was significantly reduced in all the brain regions examined at the age of 4-6 months (Fig. 4A-I). Together, these data indicate that APE1 cKO driven by the CamKIIα promoter may disrupt excitatory and inhibitory circuitry, but not the overall density of synaptic structures, at 6 months of age.

### APE1 cKO potentiates dendritic spine shrinkage and impairs electrophysiological function of the mice brain

**Figure 5.**
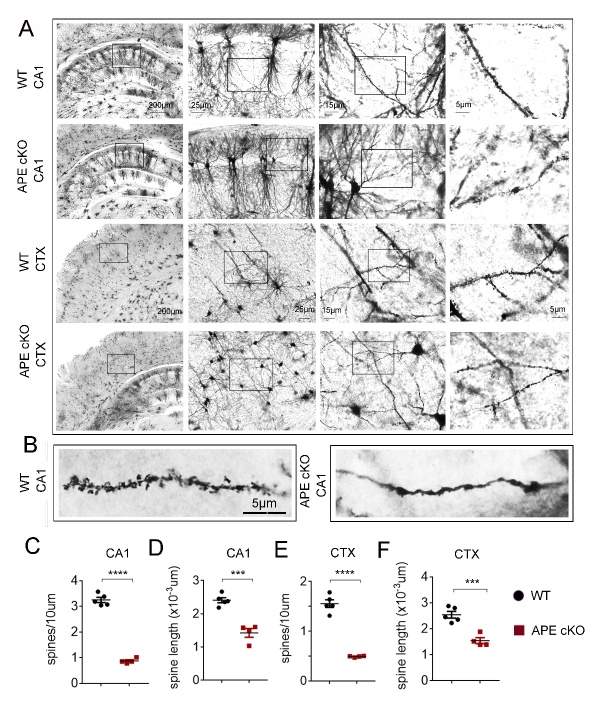
APE cKO reduces dendritic spines in hippocampal CA1 and the cortex. (A) Representative images of Golgi-stained sections of the dorsal hippocampus and cortex of WT and APE cKO mice at 6 months of age. (B-E) Quantification of number of spines per unit length and spine length in the indicated brain regions of the same animals. All data shown as as scatterplots with the mean±SEM, n=5 WT, 4 APE cKO animals/group. ***P<0.001, ****P<0.0001 analyzed by Student’s t-tests.

**Figure 6.**
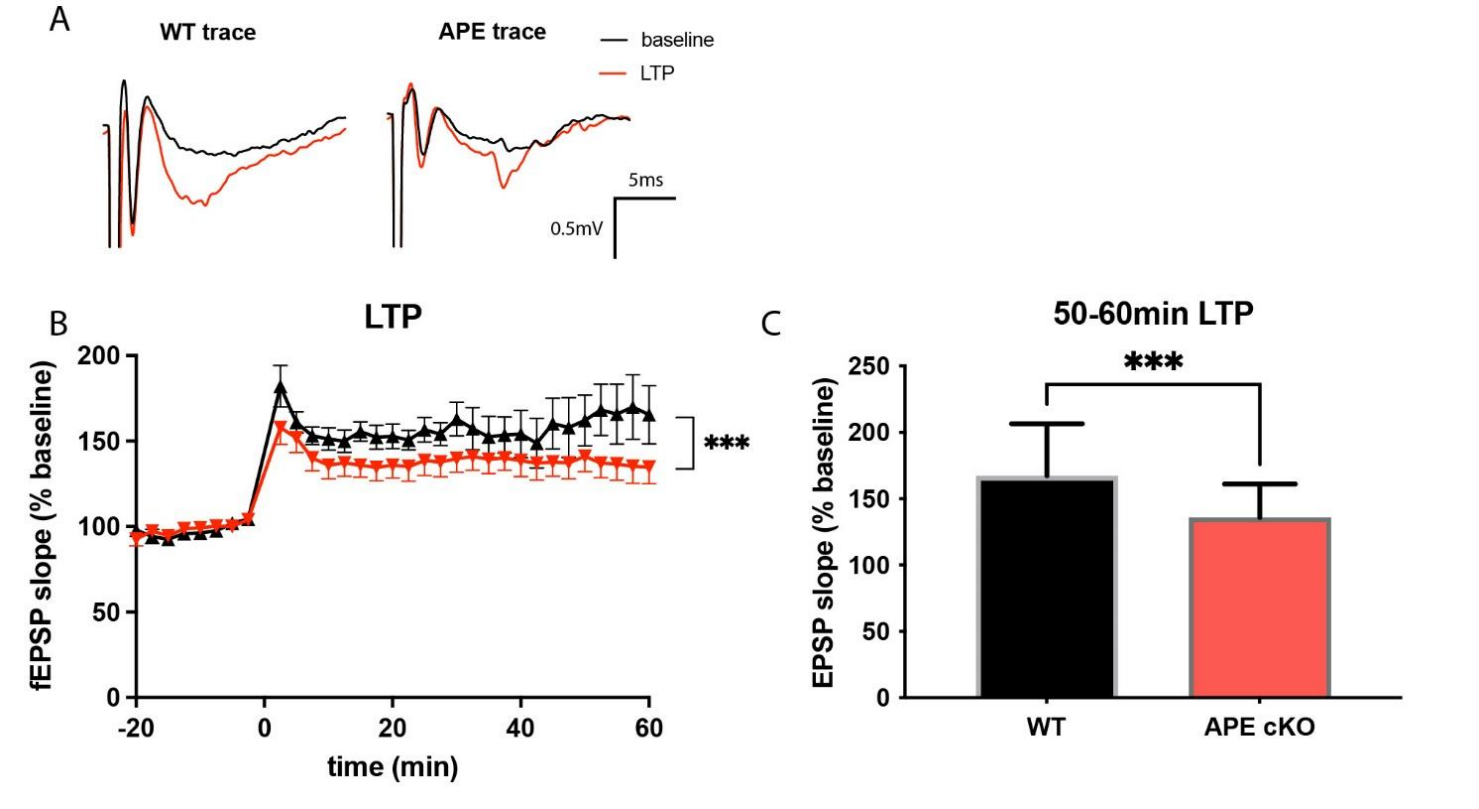
APE cKO mice showed reduced field excitatory postsynaptic potential in the hippocampal brain slice. Electrophysiological analyses of long-term potentiation (LTP) from the hippocampal region. (A) Representative traces of fEPSPs. (B) APE cKO mice showed significantly decreased level of fEPSPs after LTP induction. (C) The retention of LTP was assessed over the 50-60 min timeframe after LTP induction. n=5 WT, 7 APE cKO animals/group. Data in (B) shown as mean±SEM; data in (C) shown as mean±SD, ***P<0.001 analyzed by two-way ANOVA followed by Tukey post-hoc.

Dendritic spines are small protrusions from neuronal dendrites that are considered major sites of information processing and storage [43, 44]. To examine whether the APE1 cKO affects dendritic spines, we analyzed Golgi-impregnated neuronal structures from the mice at 4-6 months of age [43, 44]. APE1 cKO significantly reduced the number of apical dendritic spines as well as the length of these spines within both the CA1 and layers II/III of somatosensory cortical regions (Fig. 5A-E). As shown in the magnified images of the dendrites, APE1 cKO leads to significant shrinkage of dendritic spines compared to WT mice (Fig. 5F). In order to extend the above morphological findings into a functional level, we examined long-term potentiation (LTP) in the hippocampus. APE1 cKO decreased the percentage and the slope of the field excitatory postsynaptic potentials (fEPSPs) in CA1 compared to WT (Fig. 6A, B), indicating a loss of synaptic function in the APE1 hippocampus.

## DISCUSSION

Aging is characterized by an accumulation of oxidative stress [45], which can culminate in oxidative DNA damage and a resulting decline in cognitive function [46]. The enzyme APE1 has a variety of cellular functions, including oxidative DNA repair as well as transcriptional modification and cellular activity, and has been shown to facilitate post-stroke neurological recovery [26] and prevent brain tumor formation by preserving the integrity of the neural genome [47]. However, it remains unknown whether a deficiency in APE1 in neuronal cells will accelerate aging-related cognitive decline. Therefore, we generated the APE1 conditional knockout mice under the control of CaMKIIα, a promoter driving Cre recombinase expression in the forebrain, including the CA1 pyramidal cell layer of the hippocampus [29]. The present study reveals that conditional knockout of APE1 under the control of CaMKIIα leads to loss of both excitatory and inhibitory synaptic components, dendritic spine shrinkage, impaired LTP, and memory disruption between the ages of 4-6 months, well before any of these features are observed in WT brain. Thus, this conditional knockout mouse may serve as an animal model of premature neurological decline.

Synaptic connectivity between neurons is an essential component of normal cognitive function [48]. Glutamatergic synapses are particularly important for the emergence of LTP and the formation of memories. Consistent with our observation that APE cKO reduced the expresson of the vesicular glutamate transporter vGLUT1 in the termination zone of Schaeffer collaterals (CA3 and CA1), the cKO mice also exhibited impairments in LTP and memory, but not learning, aspects of the Morris water maze. This phenotype echos that of vGLUT1 knockout mice, wherein LTP is impaired despite a lack of behavior changes in the learning arm of the Morris water maze test [49]. Our observation that the novel object discrimination index was not different between mouse genotypes requires further exploration. Novel object testing can be highly sensitive to age as well as testing paradigms [50]. Our testing protocol may have included too much time after the introduction of the novel object, and thus the exploration may have been “diluted” as the mouse became quickly familiarized. We plan to shorten the length of time analyzed for the discrimination index, as well as extend the age beyond the current period of 6 months. In addition, cognitive behaviors rely on an complex network of activity, involving both compensatory systems as well as modulation by other cell types, such as myelinating oligodendrocytes. For this reason, we avoid conclusions that relate a specific brain region to a specific behavioral outcome.

Aging-induced impairments in the presynaptic and postsynaptic components of the GABAergic system also may result in cognitive dysfunction by disturbing the balance between inhibitory and excitatory neuronal activities [51]. CaMKIIα specific depletion of APE1 resulted in loss of both pre- and post-synaptic GABAergic markers (GAD and GABRA1, respectively) in all areas examined in the hippocampus and to an extent in sensorimotor cortical layers II/III. We do not expect that the loss of GABAergic markers is directly due to the loss of APE1 within inhibitory neurons, as CaMKIIα promotor activity (and thus APE1 knockout) is not associated with GAD-positive neurons [52]. Thus, further investigation to determine how potential loss of APE1 in excitatory neurons can lead to the loss of GABAergic markers is merited. The loss of GABAergic immunoreactivity has been identified as a feature of the aging brain and is associated with cognitive impairment [51, 53]. Further studies on synaptic disinhibition or inhibitory signaling in the aging brain of APE1 cKO mice would build well upon the current study.

In addition to alterations in neurotransmitter markers, aging has been closely associated with morphological alterations of neuronal synapses and dendritic spines [54]. Although we did not observe differences in the total number of synapses in our APE1 cKO brains, we observed morphological alterations in dendritic spines that are consistent with an aging phenotype and were absent in age-matched controls. The reduction in dendritic spines of the APE1 cKO mice - both in number and in length - is likely to contribute to the observed impairments in electrophysiological function as well as worsened memory function.

Aging-related cognitive decline is emerging as a critical health concern [1, 2] and poses an enormous socioeconomic burden. Animal studies of this phenomenon are challenging, as it usually takes ~12-18 months for aging-related deficits to emerge [55]. Developing a practical animal model for aging-related studies is of great importance; in this context, it is important to note that subtle morphological or electrophysiological changes may occur even before six months of age in the brains of APE1 cKO mice. However, the current study details mild neural changes consistent with the observed cognitive impairment in APE1 cKO mice that was undetected in wildtype animals. These findings increase the potential of the APE1 cKO mice as a model for premature development of an aging phenotype, thereby avoiding survivor bias in aging colonies and mimicking the endogenous processes that contribute to aged brain phenotype.

The present report shows that CaMKIIα-driven conditional knockout of APE1 in forebrain neurons forces premature phenotypic changes associated with aged adults, which may serve as a promising model for research aging-related deficits with an accelerated timeframe of 4-6 months.
